# Designing hospitality for health: an emerging approach to public health

**DOI:** 10.3389/fpubh.2026.1858623

**Published:** 2026-06-15

**Authors:** Brandon D. Howell, Lucas Steffy, Jordan R. Hill, Reid Parks, Ivaldo Silva, Edmond Ramly, Richard J. Holden

**Affiliations:** 1 School of Public Health Bloomington, Department of Health and Wellness Design, Indiana University, Bloomington, IN, United States; 2 Center for Health by Design, Indiana University School of Public Health, Bloomington, IN, United States; 3 Indiana University School of Medicine, Bloomington, IN, United States; 4 Department of Industrial and Systems Engineering, College of Engineering, University of Wisconsin, Madison, WI, United States; 5 School of Public Health Bloomington, Department of Applied Health Science, Indiana University, Bloomington, IN, United States; 6 Indiana University-Bloomington, School of Public Health, Bloomington, IN, United States

**Keywords:** experience design, healthcare, hospitality, long-term care, wellness lodging

## Abstract

Health is shaped not only by clinical care but also by the environment and interactions people experience in health spaces such as health centers, long-term care communities, and wellness-oriented lodging. This viewpoint argues for continually recognizing and developing *Hospitality for Health* as an academic focus integrating evidence-based experience design, hospitality, environmental psychology, and systems engineering. Research shows that features of health spaces such as privacy, optimized acoustics, and empathetic services can reduce stress, improve sleep, enhance satisfaction, and support safety and recovery. Viewing health spaces as interconnected restorative environments highlights opportunities for cross-sector curriculum development, research, and innovation. Further formalization of this field can strengthen public health, workforce well-being, and organizational performance, while potentially reducing health disparities, by promoting environments that support dignity, autonomy, and restoration across the lifespan.

## Introduction and rationale for *hospitality for health*

This viewpoint positions *Hospitality for Health* as an emerging, practice-informed, and comprehensive design approach with value for public health. The central premise is that health is shaped not only by clinical interventions, but also by the cumulative experiences people have while receiving care, residing in care settings, and seeking rest or recovery in hospitality and wellness-oriented lodging environments. In this context, *experience* refers to the perceptions, emotions, and interactions that shape how individuals feel and function within service environments (1–3). *Experience design* is the intentional shaping of these interactions through the intentional arrangement of physical space, service processes, communication, and cultural cues to support desired emotional, behavioral, or health outcomes (3–6).

Health centers, long-term care communities, and emerging wellness-oriented lodging environments can be conceptually understood as a continuum of care and comfort across the lifespan; although the strength of empirical evidence supporting health outcomes varies across these settings. For leaders in healthcare, long-term care, public health, and hospitality, this framing offers a strategic opportunity to address enduring challenges in quality and equity of care, resident and guest well-being, operational efficiency, and workforce sustainability (7, 8). A growing body of evidence demonstrates that both environmental conditions and service experiences significantly influence health outcomes (9–16). However, institutional settings, such as acute care units, adult day services, long-term care communities, and wellness-oriented lodging, often fall short in providing environments that feel personal, restorative, and coherent (8, 17). These environmental shortcomings can negatively shape perceptions of care quality and overall satisfaction (9). Evidence from the evidence-based healthcare design literature further shows that physical environments are far more than aesthetic considerations. Design features such as single-patient rooms, acoustic control, access to daylight and nature, and supportive spatial layouts are linked to clinically meaningful outcomes, including improved safety, reduced infections, lower stress and pain levels, better sleep quality, and shorter lengths of stay (10, 11).

Across health centers, research provides consistent evidence that poorly designed environments and interactions (e.g., impersonal communication, excessive noise, lack of privacy, and confusing layouts) are associated with higher stress and diminished satisfaction and reduced engagement among patients and families (9, 12). In long-term care, institutional routines and non-homelike design features can erode autonomy and a sense of “home,” contributing to distress and disengagement among residents (13, 14). In wellness-oriented lodging, an emerging and less empirically developed domain, hospitality and environmental psychology research links stressful environments to disrupted sleep, fatigue, and reduced restoration, reinforcing the role of experience in shaping recovery beyond formal care (15, 16).

What is worse, poor health experiences are not randomly distributed, disproportionately affecting groups that also experience medical, socioeconomic, and other disparities. For example, in one national study of patient experiences, the lowest-income patient group had 1.3–1.6 higher odds of reporting poorer access, communication, delays, and satisfaction with providers in their health encounters (18).

## Evidence and applications across health spaces

Evidence-based healthcare design demonstrates that physical and experiential features of health spaces affect more than subjective impressions of the encounter; they can influence stress physiology, sleep, safety, and recovery (10, 11). Design interventions associated with improved patient outcomes and experience include:

- Single-patient rooms, which support privacy and reduce disturbances, are associated with improved patient experience and, in some contexts, reduced infection risk (10, 11, 19).
- Acoustic design (quieter environments), which is associated with reduced stress and improved sleep (10).
- Natural light and views of nature, which are associated with decreased anxiety and depression, and in some studies, shorter length of stay (10, 20).
- Art and biophilic elements, which are associated with reduced stress and perceived pain and improved well-being (10, 21).

A systematic review of hospitality in healthcare underscores that when patients experience genuine welcome, attentiveness, and “host–guest” style care, they report stronger trust and a better overall healing experience (12, 21). Quantitatively, patient-centered environmental upgrades translate to clinical gains: in a cardiac ICU, access to daylight and window views reduced length of stay by ~16.8 h (≈1 day), with bed orientation toward the window strengthening the effect (20). At the same time, moving from multi-bed bays to single-patient rooms is associated with substantial infection reductions, meta-analyses report a 45% lower risk of healthcare-associated colonization/infection and lower bacteremia rates, underscoring safety alongside experience (11). Taken together, these findings suggest hospitality-aligned design and service can contribute to safety, stress reduction, and recovery, and thus belong within public health and evidence-informed practice (7, 10).

Hospitality organizations succeed by creating environments that feel welcoming, predictable, and personally meaningful. These principles can also inform the design of a hospital room, a long-term care apartment, or a wellness-focused hotel suite. Wellness-oriented lodging refers to accommodations designed explicitly to promote health and restoration through features such as sleep-supportive environments, stress-reduction amenities, and spaces for mindfulness, distinct from traditional hotels focused primarily on comfort and convenience (15). In each setting, individuals may be navigating vulnerability through illness, aging, or the stresses of travel.

As Jordan et al. (22) argue, incorporating hospitality and experience design into public health curricula can broaden perspectives on where and how health is created. When applied across patient, resident, and guest environments, hospitality-informed design can elevate care quality, safety, and equity by prioritizing dignity, autonomy, and emotional support (12, 22). This interdisciplinary approach encourages professionals to see medical centers, long-term care communities, and wellness-oriented lodging not as isolated silos, but as interrelated nodes in an ecosystem of the human experience, where the degree of support empirical support for health outcomes varies, but where shared experiential principles may inform cross-setting innovation.

**Figure 1:**
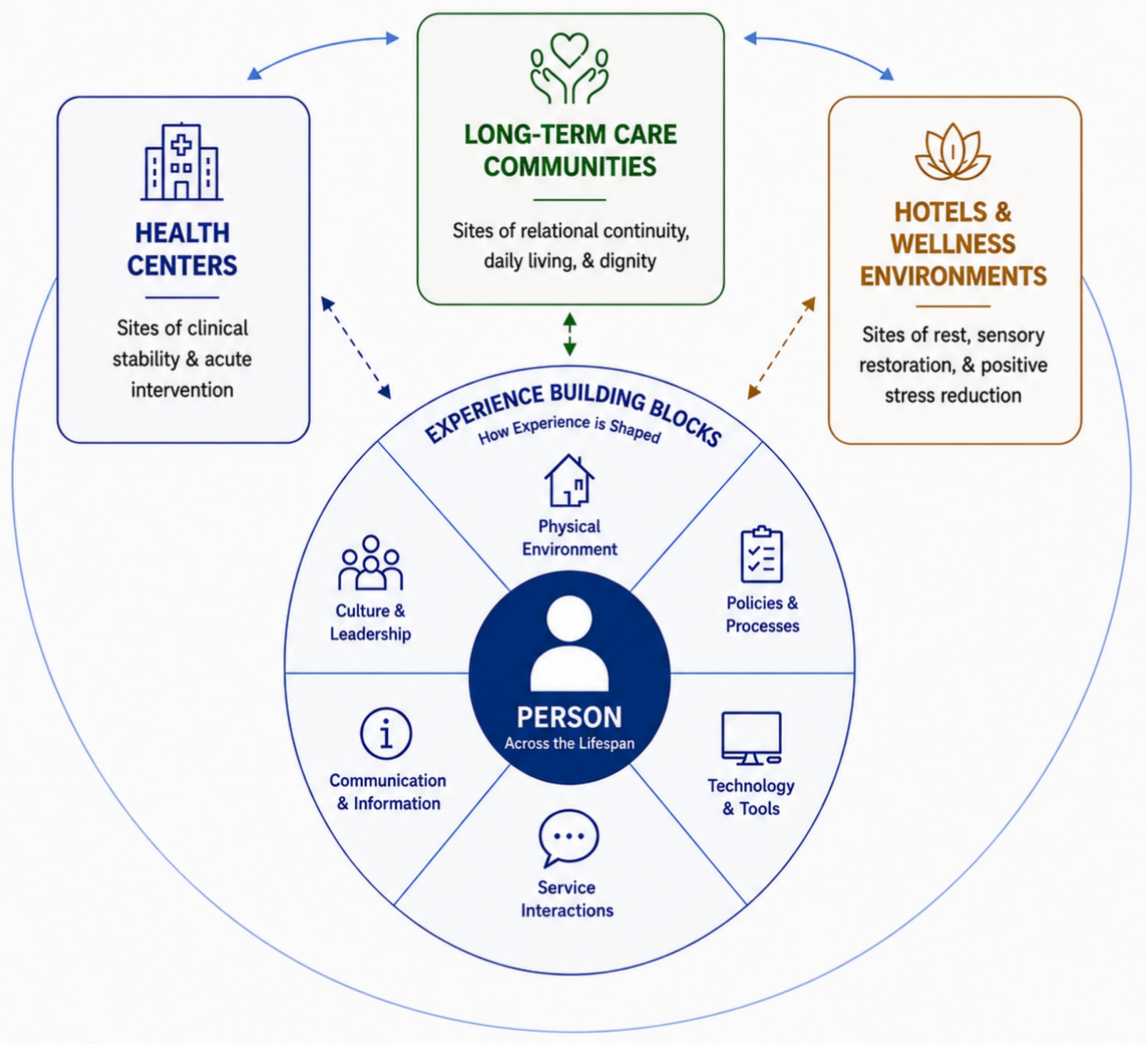
Conceptual model of health spaces whose design can shape hospitality for health.

Figure 1 depicts health centers, long-term care communities, hotels, and wellness-oriented environments as interconnected health spaces that benefit from the experience design approach inherent to *Hospitality for Health*. The central “Experience Building Blocks” model illustrates six domains that shape experience across settings. Solid arrows represent shared relationships across health spaces, while dashed arrows indicate the transfer of experience-building strategies between settings and the *Hospitality for Health* framework.

Long-term care communities represent well-established sites of care, while wellness-oriented lodging environments are an emerging domain within the broader health and wellness landscape. Residents in long-term care spend months or years in these environments, while guests in wellness-oriented lodging may seek recovery from stress, burnout, or life disruption. A person-centered and homelike long-term care design has been linked to improved mood, engagement, and sense of belonging (13, 14). In wellness-oriented lodging, restorative environments; using natural materials, daylight, quiet zones, and spaces for mindfulness, and have been associated in early and indirect research with improvements in perceived mental well-being and perceived recovery from stress (15, 16).

*Hospitality for Health* addresses a gap by integrating environments, services, systems, and human needs into universal design. It unmistakenly treats experience as a clearly designed, measurable, and moral outcome. While the Systems Engineering Initiative for Patient Safety (SEIPS) offers a core human factors framework, *Hospitality for Health* builds on this rigor but moves beyond harm prevention to ask how systems can be designed to feel supportive and humane across patient, resident, and guest journeys. Furthermore, environmental psychology and evidence-based design links physical environments to stress, cognition, recovery, and satisfaction; explaining *how* environments impact people, yet *Hospitality for Health* shapes how spaces are humanely lived.

To distinguish Hospitality for Health more clearly from adjacent fields, the following table provides a comparison across cited frameworks, highlighting its integrative and cross-setting comparison.

**Table 1:** 

Dimension	Hospitality for Health	Evidence-based design (EBD)	Human factors engineering	Environmental psychology	Hospitality management
Primary aim	To design experiences that support health, dignity, and restoration across settings	To improve clinical and operational outcomes through physical environment design	To optimize safety, performance, and system functioning	To explain how environments influence perception, cognition, and behavior	To deliver satisfaction, loyalty, and memorable guest experiences
Scope	Cross-setting ecosystem (health centers, long-term care, wellness-oriented lodging)	Primarily health center environments	Health center work systems and processes	Broad (natural and built environments, including health centers)	Hospitality and tourism contexts
Core contribution	Integrates environment, service, and experience into a unified public health-oriented framework	Provides empirical links between design features and health outcomes (10, 11)	Models interactions among people, tasks, tools, and environments to improve outcomes (23, 24)	Explains mechanisms such as stress reduction and restoration (9, 17)	Service excellence, individual needs, and experience delivery (25)
Role of experience	Central outcome that is intentionally designed and measured (1–3, 12)	Secondary outcome associated with improved environments	Emergent from system performance but not primary focus	Core explanatory construct	Core product and value driver
Distinctive contribution	Positions experience as a public health lever across a continuum of care, residence, and lodging (the human experience)	Strengthens evidence-informed design decisions in health centers	Strengthens systems-based safety, effectiveness, and efficiency	Explains environmental influences on human well-being	Advances service quality and guest engagement without a primary health focus

Regarding hospitality science, *Hospitality for Health* shifts from business market differentiation to a public health relevant practice. Ultimately, *Hospitality for Health* is distinguished based on its focus on the experience of ecosystems across the continuum of care and comfort. *Hospitality for Health* helps connect SEIPS, environmental psychology, evidence-based design, and hospitality management.

Across these settings, the evidence base is not evenly distributed; robust empirical literature exists for health centers and long-term care facilities, while wellness-oriented lodging (i.e., hospitality) represents a developing area with more limited and indirect evidence linking design to health outcomes.

### *Hospitality for health* as a lever for better healthcare for all, now

*Hospitality for Health* can support better healthcare for all by treating equitable care not only as access to services, but also how people are welcomed to, oriented within, and respected by the health spaces they access. Evidence suggests that health spaces designed for comfort, dignity, and restoration can improve both patient experience and clinical outcomes (10, 11), indicating that humanely designed health spaces can concurrently address all elements of the Triple Aim: improving patient experience, reducing population-based health disparities, and reducing cost. However, not all health spaces are designed with equal attention to hospitality, suggesting that certain patients or groups of patients will disproportionately experience confusion, sensory overload, inaccessibility, or cultural discordance. *Hospitality for Health* in contrast is a lever for improving the social, environmental, and organizational conditions that allow people to be healthy in any health space (7). This equity-centered approach also strengthens clinician-patient relationships by creating conditions for trust and open communication (26). *Hospitality for Health* obliges healthcare professionals to treat patients more holistically—meeting them medically, culturally, educationally, and emotionally—rather than reductionist treatment of patients as diagnoses, room numbers, or workflows. Such relational reframing matters for clinicians as well as patients. Healthcare workers experience burnout and psychological distress at high rates, and workplace interventions that improve well-being and humane engagement can reduce clinician burnout and strengthen the work environment (27).

However, it must be cautioned that any novel approach risks what are called intervention-generated inequalities, or the inadvertent exacerbation of outcome disparities because of differential access to, quality of, or adherence to interventions (23). For example, mass media campaigns may widen gaps between higher and lower educated individuals (28), or technology interventions may selectively benefit those with greater technology access and literacy (24). *Hospitality for Health*, too, could widen the gaps between individuals who can afford or are served by hospitable healthcare organizations versus their counterparts. Additionally, to the extent that experience is an intersubjective outcome, not all hospitality approaches will be equally positive for all, requiring that *Hospitality for Health* embrace tailoring and personalization strategies.

## Advancing education, practice, and collaboration

Formalizing *Hospitality for Health* as an academic focus would allow educators and practitioners to:

- Develop interdisciplinary training programs connecting the health center experience, long-term care community design, and lodging experience design, integrating public health, hospitality, environmental psychology, and management (15, 22).
- Embed experiential learning in health centers, long-term care communities, and lodging environments, using real-world data to explore how design and service influence stress, satisfaction, and health outcomes (10, 29).
- Train professionals including frontline clinicians, long-term care leaders, designers, hoteliers, and experience officers in empathy-driven service, evidence-based design, and experience mapping (12, 25).
- Align with Council on Education for Public Health (CEPH) competencies through cross-disciplinary collaboration and systems thinking (17, 22).

Such academic focus would prepare graduates to work fluidly between sectors, recognizing parallel challenges and opportunities in patient rooms, resident living spaces, and guest rooms, and understanding that all three can function as health-supporting environments.

A *Hospitality for Health* initiative can serve as connective tissue across sectors that rarely plan together but serve overlapping populations. Opportunities include:

- Curriculum and faculty partnerships: Co-developed courses and guest lectures spanning healthcare, long-term care, hospitality, design, and public health (22).
- Living labs: Health centers, long-term care communities, and wellness-oriented lodging properties used to pilot and evaluate design interventions such as redesigned arrival experiences, room prototypes, or restorative common spaces (10, 13, 29, 30).
- Industry and community collaborations: Partnerships with architects, interior designers, technology firms, and operators to prototype health-promoting environments and services (31).
- Shared technology and data: Joint exploration of digital wayfinding, pre-arrival communication, and personalized information to reduce anxiety before, during, and after stays (16, 31).

These collaborations can accelerate knowledge transfer: what is learned about stress reduction in a redesigned hospital unit can inform memory-care community design; insights from wellness lodging can be translated into staff respite spaces in hospitals and long-term care.

## Research agenda and call to action

Formalizing *Hospitality for Health* also invites a cross-setting research agenda. Formal systematic reviews need to be conducted to formally survey and appraise the literature, beyond the scope of the present rapid and non-systematic review. Such a review will identify gaps and future research directions. However, we preliminarily offer several research questions predicated on our review of the literature:

- In health centers, how do hospitality-aligned changes, quieter units, private rooms, nature views, or redesigned arrival and discharge experiences affect stress, pain, length of stay, and satisfaction (10, 12)? Epidemiological (e.g., case–control) and experimental (e.g., clinical trial) methods can investigate these effects’ magnitude and causal direction, respectively (40).
- In long-term care, how do home-like layouts, social spaces, and service rituals influence residents’ mood, engagement, behavioral symptoms, and functional status? Observational longitudinal research with self-reported, expert rated, and objective measurement can assess how outcomes vary by location (41).
- In wellness-oriented lodging, which design features most effectively support mental restoration and sleep, and how might these features be adapted for health centers and long-term care (15, 16)? Multicomponent interventions using step-wedge, interrupted time series, and multiphase optimization strategy frameworks can help unpack the effects from multiple features (42–44).
- Across settings, can unified experience design frameworks reduce burnout, enhance staff experience, and improve organizational outcomes such as safety, satisfaction, and retention (25, 32)? Implementation of research methods and evidence-based logic models can estimate linkages between design, outcomes, and mediating mechanisms (45, 46).
- Do *Hospitality for Health* initiatives promote intervention-generated inequalities when their design, adoption, implementation, or use privileges some groups over others? (43) Under what circumstances do hospitality interventions reduce versus exacerbate health disparities?

Addressing these questions aligns with Public Health 3.0 and other calls for cross-sector, system-oriented approaches to health and equity (7, 8).

## The frontier of hospitality for health

Health centers, long-term care communities, and wellness-oriented lodging operations across settings and client bases often share common pain points: burnout and turnover among frontline staff, service dissatisfaction among individuals served and their families, fragmented training focused on tasks rather than experience, and siloed improvement efforts targeting metrics but not underlying environments. In *Hospitality for Health*, experience design offers a unifying framework for addressing these health space challenges at the population level.

Various disciplines already working to improve healthcare can contribute to *Hospitality for Health*. For instance, human factors and systems engineering have long been involved in driving healthcare improvements, from optimizing processes to improving patient safety to reducing treatment disparities. From the human factors and systems engineering lens, health spaces are simply one type of system that can be designed to produce a variety of health-related outcomes, from financial to experiential. Human factors and systems engineering frameworks such as the Systems Engineering Initiative for Patient Safety (SEIPS) model, for example, can help identify work system components such as people, environments, tools, and tasks, whose interaction can be optimized to produce experience outcomes (29, 30). The subdiscipline of macro ergonomics similarly helps design organizational characteristics to achieve similar open-ended goals including performance and well-being (33). Thus, *Hospitality for Health* can be an interdisciplinary pursuit that leverages existing models, methods, and solutions.

Regardless of disciplinary lens, applying evidence-based hospitality concepts enables organizations to move beyond isolated “patient experience” or “guest satisfaction” initiatives and toward a systems-oriented design approach in which environments, processes, and human interactions are aligned to support well-being, performance, and resilience across the entire care and service ecosystem.

Box 1 **Examples of Hospitality-informed Practice in Healthcare**

To illustrate feasibility beyond conceptual argument, several large health organizations and partners have adopted hospitality-oriented practices; including themed, comforting environments, concierge navigation, formal service excellence training, and systemwide communication models. (These examples are offered as practice signals and case studies rather than as outcome evaluations and should not be interpreted as scientifically supported in the absence of peer reviewed evidence).

- The Walt Disney Company: A five-year, $100 million commitment to reimagine children’s hospital experiences using storytelling, characters, and design touchpoints (e.g., murals and interactive elements) to reduce stress for patients and families (Source: The Walt Disney Company press release (34).
- Cleveland Clinic: “Patients First” culture and the Communicate with H.E.A.R.T.® service excellence model to standardize empathic communication and service recovery behaviors.
  (Source: Cleveland Clinic website (35) and peer-reviewed publication (36)).
- Mayo Clinic: Complimentary concierge services to help patients and families navigate travel logistics, directions, and practical need reducing burden during complex care journeys.
  (Source: Mayo Clinic website (37)).
- MD Anderson–affiliated oncology environments: Hospitality-inspired interior design strategies (e.g., calming, and welcoming environments and positive distraction through art) described in oncology facility design case reports and major cancer campus design narratives.
  (Source: Peer-reviewed publication (10) and University of Texas Health website (38)).
- Ritz-Carlton Leadership Center: Healthcare-focused training and advisory services that translate Ritz-Carlton “Gold Standards” into patient experience, culture design, and service excellence practices for hospitals.
  (Source: Ritz-Carlton Leadership Center website (39)).

In summary, *Hospitality for Health* is a pragmatic, evidence-informed approach to strengthening public health by designing the environments where people receive care, live in community, and recover through rest. Evidence-based design shows that hospitality-aligned environments in health centers are linked to reduced stress and improved experience and, in some contexts, to safety and recovery outcomes (10, 12). When similar principles are extended to long-term care, and more tentatively to wellness lodging, they contribute to a broader continuum of environments that may support autonomy, dignity, and mental well-being across the lifespan (10, 13, 14, 16).

Formally recognizing this interdisciplinary space can prepare professionals fluent in both clinical and experiential excellence; promote joint curriculum and research initiatives that connect patient, resident, and guest experience; support shared evaluation frameworks that measure health and wellness outcomes across settings; and strengthen organizational cultures through empathy, dignity, and human connection (7, 22, 32). As systems of care and hospitality evolve, *Hospitality for Health* offers a shared language and mission: to design environments that sustain human flourishing wherever people receive care, live in community, or seek restorative rest.

## Data Availability

The original contributions presented in the study are included in the article/supplementary material, further inquiries can be directed to the corresponding author.
